# Mitral Regurgitation and Left Ventricular Outflow Tract Obstruction: Confluence of Challenges for Transcatheter Treatment

**DOI:** 10.31083/j.rcm2504134

**Published:** 2024-04-03

**Authors:** Antonio Sisinni, Manuel Barreiro-Pérez, Francisco Calvo-Iglesias, Rodrigo Estévez-Loureiro

**Affiliations:** ^1^Department of Cardiology, University Hospital Alvaro Cunqueiro, 36312 Vigo, Spain; ^2^Cardiovascular Research Group, Department of Cardiology, University Hospital Alvaro Cunqueiro, Galicia Sur Health Research Institute (IIS Galicia Sur), Servizo Galego de Saude, University of Vigo, 36312 Vigo, Spain

**Keywords:** mitral regurgitation, transcatheter therapies, left ventricular outflow tract obstruction, management

## Abstract

The intricate interplay between mitral regurgitation (MR) and left ventricular outflow tract (LVOT) obstruction may result in two clinical scenarios: prosthesis-related LVOT obstruction after mitral valve replacement (TMVR) and systolic anterior motion (SAM)-associated MR. This review provides a comprehensive overview of the pathophysiology, risk assessment, and transcatheter interventions for mitigating the likelihood of LVOT obstruction in patients undergoing TMVR. In addition, it extends its focus to SAM-associated MR, elucidating the different aetiological mechanisms contributing to this phenomenon, beyond hypertrophic cardiomyopathy. Transcatheter treatment options, are explored as potential therapeutic strategies, offering insights into their hemodynamic effectiveness and limitations.

## 1. Introduction

Mitral regurgitation (MR) and left ventricular outflow tract (LVOT) obstruction represent two distinct cardiac conditions, with different underlying etiological mechanisms, involving separate areas of the heart. However, the two entities can sometimes have direct connections and impact each other due to the complex interplay within the heart chambers and valves. In this perspective, two different clinical scenarios can be identified, namely (a) prosthesis-related LVOT obstruction after mitral valve (MV) replacement (TMVR) and (b) systolic anterior motion (SAM)-associated MR. Transcatheter strategies involving single or multiple devices, had been developed to treat both conditions, in single or staged procedures, in patients with high risk for surgery. In this review, we aim to discuss the pathophysiology of the interaction between MR and LVOT obstruction, focusing on transcatheter options to treat or to prevent development of outflow obstruction in patients with significant MR.

## 2. Mechanisms of Transcatheter Prosthesis-Related LVOT Obstruction

With the rise in the population of patients deemed unfit for traditional surgery and seeking alternative therapeutic avenues, there has been a surge in interest regarding TMVR, including mitral valve-in-valve (ViV), valve-in-ring (ViR), or valve-in-mitral annular calcification (ViMAC). Various devices are presently undergoing investigation within this context [1].

A significant challenge posed to TMVR pertains to the potential risk of LVOT obstruction. This complication arises from the displacement of the native or bioprosthetic anterior mitral leaflet (AML) toward the LVOT by the transcatheter valve and appears to stem from the complex interplay of various elements. These factors encompass either the inherent native anatomy or the characteristics of the surgical bioprosthesis or annuloplasty ring. First, the aortic and mitral valves are connected by fibrous continuity, and when a prosthesis is implanted at the mitral annulus, it extends into the left ventricular (LV) cavity, closely neighboring the aortic annulus. The aorto-mitral-annular (AMA) angle, representing the angle between the aortic and mitral annular planes, influences the degree of prosthesis protrusion into the LVOT. A smaller AMA angle, closer to 90°, increases the risk of LVOT obstruction. In addition, a small LV cavity, septal hypertrophy and asymmetric bulging (*fixed* LVOT obstruction risk), redundancy of the anterior (*dynamic* LVOT obstruction risk) or posterior mitral leaflets must be taken into account. Moreover, hypercontractile ventricle and atrial fibrillation can contribute to LVOT obstruction following MV replacement. Concerning surgical valve features, the angle and depth of implantation as well as height and type of leaflets, with pericardial ones potentially resulting in more pronounced LVOT obstruction, should be considered. Finally, the selection of a previous annuloplasty ring can also influence the extent of obstruction in ViR procedures, since the presence of a semi-rigid or flexible ring might elevate the likelihood of outflow tract obstruction [2], meanwhile a rigid annuloplasty rings might increase the risk of residual paravalvular leak after TMVR.

## 3. Pre-Procedural Evaluations to Assess the Risk of LVOT Obstruction

**Fig. 1.**
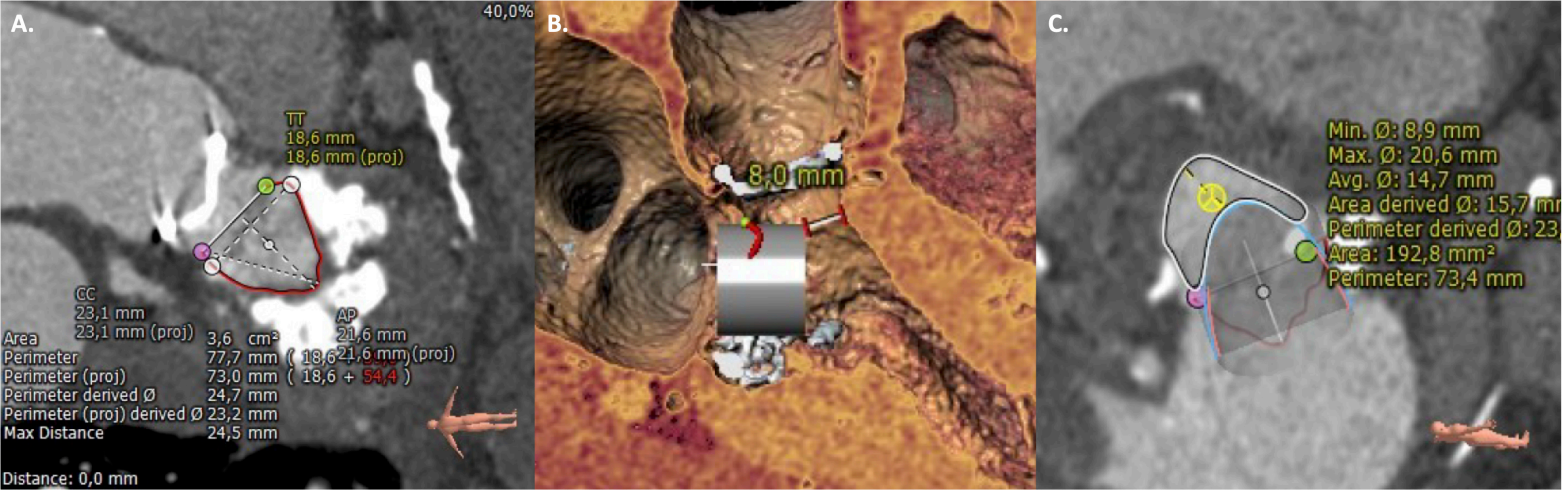
**Valve-in-mitral annular calcification (ViMAC) procedural planning**. Contrast-enhanced computed tomography (CT) scan depicting a posterolateral 180° extension mitral annular calcification (A). Three-dimensional virtual valve implantation (B). Neo-left ventricular outflow tract (LVOT) area based on virtual valve position (C). Adapted from: Barreiro-Perez M, *et al*. J. Clin. Med 2021, 10: 5973 [4].

In patients being considered for TMVR, a gated cardiac computed tomography (CT) assessment is required for determination of anatomical suitability to procedure. Given the excellent spatial resolution, CT imaging allows for an adequate assessment of the LVOT anatomy and a clear definition of LV structures, to quantify the likelihood for LVOT obstruction, based on the neo-LVOT area. The concept, proposed by Blanke *et al*. [3], relies on the extension of the outflow tract into the LV occurring after TMVR. Before procedure, LVOT is limited by the basal septum, the intervalvular fibrosa, and the basal section of the AML. In the context of TMVR, AML or previous bioprosthetic leaflets are septally deflected by the TMVR device, leading to an elongation of the outflow tract toward the LV, termed neo-LVOT, meanwhile the native LVOT at the level of the intervalvular fibrosa remains unchanged. Of note, neo-LVOT lies in a different anatomic axis than the native outflow tract. Through computer-aided design simulations, after segmentation of the mitral annulus, bioprosthetic sewing or annuloplasty ring, and measuring baseline LVOT area during systole, when the cavity is smallest, a virtual valve is then superimposed onto the annulus, allowing measurement of the neo-LVOT area (Fig. 1, Ref. [4]). Proof-of-concept validation was performed by Wang *et al*. [5] in a series of 38 patients undergoing TMVR with compassionate use of balloon-expandable valves. The predicted neo-LVOT surface area prior to the procedure exhibited a strong correlation with post-TMVR measurements and receiver operating curve identified a neo-LVOT surface area cut-off value of ≤189.4 ${\mathrm{mm}}^{2}$. This threshold demonstrated a sensitivity of 100% and a specificity of ~97% in predicting a post-TMVR increase in LVOT gradient of 10 mmHg or more, corresponding to the definition of iatrogenic LVOT obstruction according to Mitral Valve Academic Research Consortium criteria [6]. Additional measurements, like the skirt neo-LVOT, can be performed to predict the area if the AML doesn’t cover the open cells of the transcatheter valve stent frame [7]. It is worth mentioning that these predictions can vary with different TMVR devices. When compared to a cylindrical balloon-expandable valve, the predicted neo-LVOT for low-profile transcatheter valves such as Tendyne (©Abbott Vascular, Santa Clara, CA, USA) and Intrepid (©Medtronic, Minneapolis, MN, USA) will generally show a slightly larger size, because of minimized stent frame projection into the outflow tract.

In the Mitral Implantation of Transcatheter Valves (MITRAL) trial high-risk patients with severe symptomatic MV disease (either regurgitation or stenosis) due to failure of mitral surgical bioprosthesis [8] or ring annuloplasty [9] or mitral annular calcification (MAC) [10] underwent balloon-expandable transcatheter SAPIEN XT or SAPIEN 3 valve (©Edwards Lifesciences, Irvine, CA, USA) implantation. Interestingly, screen failure rate related to the risk of LVOT obstruction was ~30%, considerably higher in the ViMAC cohort with almost half of patients excluded.

Nevertheless, there are still limitations in predicting neo-LVOT, mainly related to interobserver variability in valve positioning and landmark measurements. In addition, unpredictable procedural issues might influence patients’ outcome in terms of outflow tract obstruction. In the MITRAL trial, although meticulous screening and active measures to reduce the risk of LVOT obstruction, ~10% ViMAC patients experienced that complication with hemodynamic compromise, meanwhile no significant outflow gradient increase was noticed in ViV and ViR groups [11]. In the multicentre CHoice of OptImal transCatheter trEatment for Mitral Insufficiency Registry (CHOICE-MI), high-surgical risk patients with MR, considered suboptimal candidates for mitral transcatheter edge-to-edge repair (M-TEER), underwent TMVR with 10 dedicated devices. Except for annular dimensions that were not within the available treatment ranges, the substantial rate of screening failures was primarily attributed to the risk of outflow tract obstruction. Nonetheless, acute LVOT obstruction occurred in ~4% of patients and, between periprocedural complications, was the one with the strongest association with 2-year mortality (hazard ratio (HR) 4.03, 95% confidence interval (95% CI): 1.46–9.10; *p* = 0.01) [12].

## 4. Transcatheter Strategies for Mitigating the Likelihood of LVOT Obstruction

Distinct percutaneous approaches have been suggested in the perspective of a proactive prevention of extremely probable LVOT obstruction immediately after TMVR, as an alternative to (a) excluding patients as candidates for MV replacement or (b) isolated medical management of outflow tract obstruction-related hemodynamic compromise. These procedures differ according to the timing (staged *vs*. concomitant) and the target (AML *vs*. basal septum).

Laceration of the Anterior Mitral leaflet to Prevent Outflow ObstructioN (LAMPOON) is a transcatheter electrosurgical procedure involving the intentional laceration of the $A_{2}$ segment of AML using a coronary wire and radiofrequency ablation, immediately before performing TMVR. In the first-in-human experience, 5 patients with MV disease and prohibitive risk for LVOT obstruction underwent successful and uncomplicated LAMPOON procedure, resulting in only a slight increase in LVOT gradients after subsequent TMVR [13]. The LAMPOON investigational device exemption trial was a prospective multicenter study, enrolling 30 patients equally distributed between native MAC and previous mitral annuloplasty ring, currently not suitable for surgical treatment, showing primarily MR in one third of cases, with a high risk of fixed (n = 25) or dynamic (n = 5) LVOT obstruction. Patients were submitted to LAMPOON procedure before SAPIEN 3 valve implantation. Procedural success was achieved in all subjects, even though 27% of patients required additional interventions before leaving the catheterization laboratory, to treat paravalvular leak (PVL) or decrease high skirt neo-LVOT gradients, observed before protocol amendment. Nevertheless, the overall 30-day follow-up survival was 93%, significantly higher when compared to ~50% survival after ViMAC-related LVOT obstruction in MITRAL trial [14]. Since the initial combined antegrade and retrograde approach requiring creation of an arteriovenous rail was complex, an antegrade-only transseptal technique has been developed. The simplified method involves using two 6F guiding catheters, usually JR4 and MP, the latter of which guides an electrified guidewire (Astato wire, ©Asahi Intecc, Nagoya, Japan) within an insulated Piggyback Wire Converter (©Teleflex Medical, Wayne, PA, USA) perforating the center and base of the AML. Positioned in the LV, the JR4 catheter carries a snare used to capture the electrified guidewire’s end. The guidewire is then pulled out to create a loop, which is electrified to lacerate the AML along the centerline from base to tip. Consequently, as the AML moves towards the LVOT, this division of the leaflet causes the two halves to separate, effectively preventing obstruction of the outflow tract [15]. In the first experience, the simplified technique was effective in all 8 subjects enrolled and resulted in a significantly reduced procedural time, compared with the retrograde technique in the LAMPOON investigational device exemption trial (39 ± 9 *vs*. 65 ± 35 min) [16].

The Balloon-Assisted Translocation of the Mitral ANterior leaflet (BATMAN) is a hybrid procedure, performed on cardiopulmonary bypass, through advancement of a pericardiocentesis needle via transapical access through the AML into the left atrium (LA). After exchanging the needle stylet with a 0.035” stiff wire, a 20-mm valvuloplasty balloon is placed over the wire, positioned within the anterior leaflet, and then inflated, creating a large defect, or hole, in the body of AML, where valve prosthesis is then deployed via the transapical access. In that way, BATMAN procedure could potentially mitigate the risk of displacing the bulky AML into the LVOT and allow for a sealing effect to reduce the occurrence of PVL. This approach had been performed on 3 patients at high risk of LVOT obstruction before SAPIEN 3 positioning, with procedural success in all cases. Limitations of the technique rely on less controlled splitting of the AML, compared to LAMPOON, and transapical approach. In this perspective, a modified transseptal version, not requiring cardiopulmonary bypass, is currently under investigation [17].

TMVR device with a specific design to prevent anterior leaflet displacement had been developed. The SAPIEN M3 system (©Edwards Lifesciences, Irvine, CA, USA) is a fully transseptal TMVR system delivered through a 28-F femoral introducer, comprising two distinct components: the dock and the valve. The balloon-expandable valve mirrors the 29-mm diameter SAPIEN 3 aortic valve. Constructed from nitinol and coated with polytetrafluoroethylene, the dock is recapturable, repositionable and specifically engineered to encircle the chordae tendineae below the mitral annulus, beginning with a larger diameter (37 mm) leading turn. Subsequent turns present a smaller diameter (25.5 mm) and provide a landing zone for the valve, while their covering aids in preventing migration or embolization [18]. The ongoing ENCIRCLE trial (NCT04153292) is recruiting patients with MR ≥3+, New York Heart Association ≥II, considered unsuitable for commercial options as assessed by heart team, to undergo TMVR with SAPIEN M3 system.

The HighLife system (©Highlife SAS, Irvine, CA, USA) is a dual component system consisting of the valve itself and a sub-annular implant ring. The sub-annular implant is a polymer tube, coated with polyester, that encompasses a nitinol hook, aiming to create a complete ring encircling the mitral sub-annular apparatus, through two distal ends situated on either side of a previously positioned guidewire loop. The first end tapers with a nitinol clip, while the second end flares to accommodate the clip. As the open ring is advanced along the guidewire loop, the two ends come into proximity until the clip securely connects with the opposing end, effectively sealing the ring. The second component, the valve, comprises a nitinol self-expanding frame, covered by a polyester graft, accompanied by three porcine pericardial leaflets. The frame configuration includes a preformed groove in the annular region, ensuring optimal contact with the previously placed sub-annular implant. In terms of procedure, the sub-annular implant is introduced using an 18-F catheter via the femoral artery, and retrogradely advanced through the aortic valve over the previously positioned guidewire loop. Subsequently, the valve is introduced via femoral vein through a 39-F catheter delivery system, positioned to facilitate complete deployment of the prosthetic valve’s outflow within the ventricle, distal to the sub-annular implant. The valve’s outflow is then manually pushed towards the LA until achieving close contact with the sub-annular groove. Ultimately, the inflow end of the transcatheter mitral valve is deployed. The sub-annular implant together with the native leaflets may provide complete paravalvular sealing [19, 20]. The HighLife valve is offered in two variations: the standard valve and the open-cell Clarity valve, which is specially designed for patients at risk of LVOT obstruction. No data are currently available on the Clarity system, however the HighLife Trans-septal Mitral Valve Replacement Feasibility Study of the Open Cell CLARITY Valve (HighFLO) study (NCT04888247) will provide a deeper understanding of the feasibility and initial results linked to this device in that subset of patients.

**Fig. 2.**
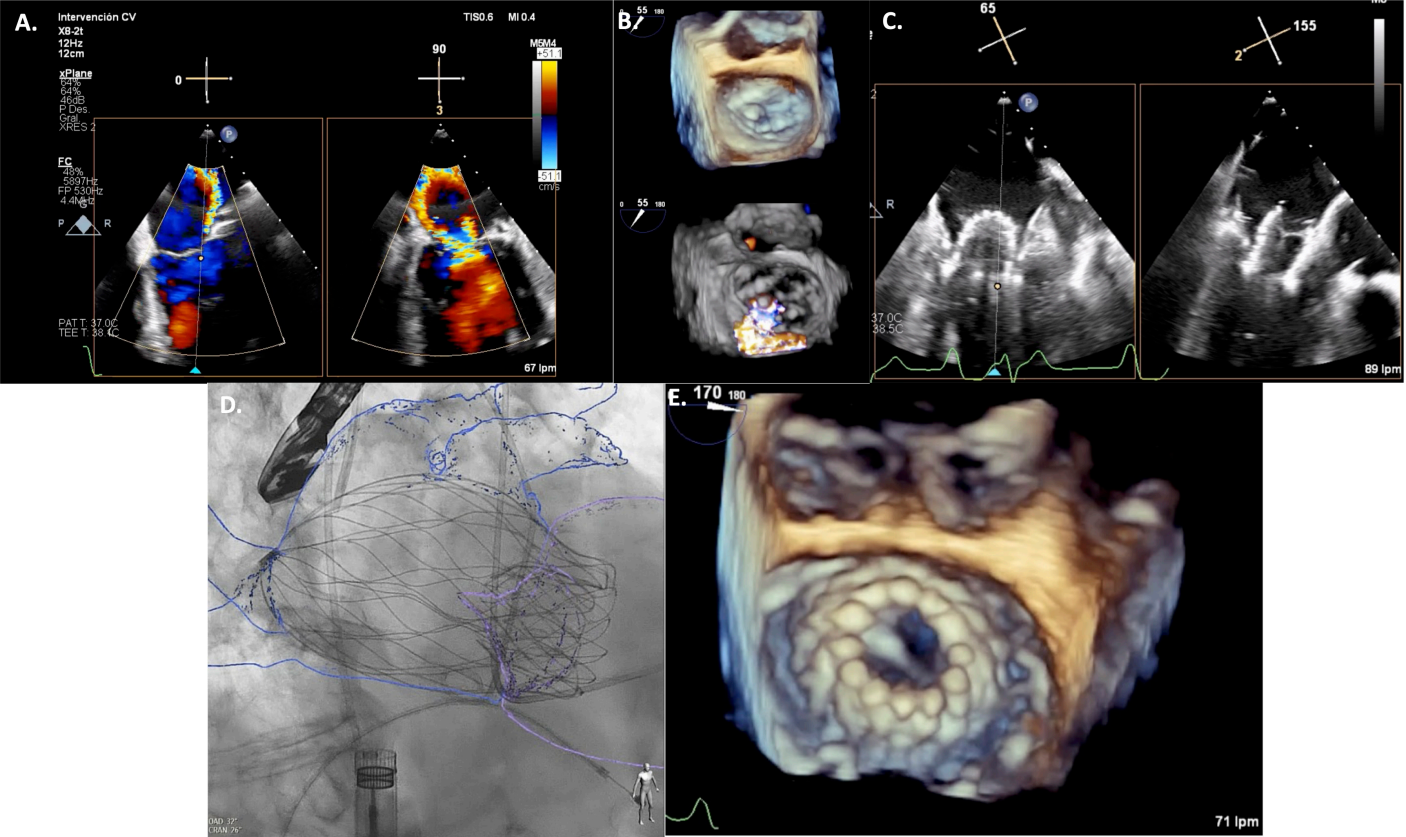
**AltaValve implantation procedure**. Transoesophageal echocardiogram showing baseline severe mitral regurgitation (MR) (A and B). Intraprocedural echocardiography guiding AltaValve implantation (C). Final fluoroscopic and echocardiography revealing adequate device positioning (D and E, respectively).

Although not specifically designed for patients at risk for LVOT obstruction, the AltaValve (©4C Medical Technologies, Minneapolis, MN, USA) overcomes that issue via the supra-annular atria-only fixation technology. Device consists of a self-expanding nitinol frame with a spherical configuration, which is sized between 50 and 95 mm, housing a 27-mm tri-leaflet bovine pericardium valve, hydrodynamically equivalent to a 29-mm surgical valve. Additionally, a fabric skirt is added to the lower part of the frame, serving as an annular ring, to inhibit the occurrence of PVL [21]. The dimensions of the implant ball are oversized, ranging from 10% to 30% beyond the measurements of the LA, meanwhile the desired oversizing for the annular ring ranges from 5% to 20%, calculated in relation to the maximum MV diameter. Currently, the AltaValve is available with three different annular ring sizes: 40 mm, 46 mm, and 54 mm in diameter. After achieving access to the LA, via either a transeptal or transapical approach, the valve is loaded and gradually unfolded as the delivery catheter is retracted. AltaValve is repositionable and, interestingly, fully recapturable even after complete deployment, leading to improved procedural safety [22]. Design of the AltaValve does not require pre-procedural neo-LVOT evaluation and, in addition, aims to reduce other potential complications that can occasionally arise with other TMVR technologies, including device embolization and LV dysfunction related to device interaction with subvalvular structures. The stent of the AltaValve also permits future access to the LA for additional procedures (Fig. 2). Finally, among its unique features, the AltaValve is specifically developed for treating patients who have undergone previous transcatheter MV repair. Proof of this, in the AltaValve Early Feasibility Study Protocol (NCT03997305), evaluating the safety and performance of the system for the treatment of patients with severe MR, prior surgical MV repair, annuloplasty, or MitraClip are not considered as exclusion criteria, unless interference with AltaValve placement is likely.

Alcohol septal ablation (ASA) was initially introduced during the 1980s as a therapeutic approach for hypertrophic cardiomyopathy (HCM) [23]. The percutaneous technique involves cannulating the left main artery using a guiding catheter, advancing a 0.014” wire and an over-the-wire balloon into the septal perforator branch supplying the basal anterior septum. Balloon is then inflated to occlude blood flow. Selective angiography is conducted through the balloon’s lumen to verify accurate positioning, absence of collateral flow, and prevention of backflow into the left anterior descending artery. Additionally, microbubble contrast is introduced while performing simultaneous transthoracic echocardiography, ensuring that the interventricular septum is suitably opacified in the intended area without causing opacification of unwanted structures like papillary muscles. Finally, slow infusion of 0.5 to 1 mL of 98 dehydrated ethanol is performed to induce septal artery occlusion, which is then angiographically confirmed [15]. Up to 25% patients may experience transient or permanent complete heart block within 72 hours of the procedure [24]. The application of ASA was first documented as an effective treatment for addressing LVOT obstruction subsequent to MV annuloplasty [25]. A subsequent small series of patients who underwent alcohol septal ablation subsequent to ViMAC interventions also exhibited immediate improvements in gradient measurements post-procedure [26]. Presently, there is growing enthusiasm for conducting this procedure prior to TMVR for patients considered to have an elevated risk of developing LVOT obstruction after the intervention, translating ASA from a bail-out to a prophylactic strategy. In the first-in-man study, 20 patients with severe MV disease underwent pre-emptive ASA to mitigate TMVR-induced LVOT obstruction risk. Forty-day post-procedural CT imaging revealed a 111.2 ${\mathrm{mm}}^{2}$ (interquartile range 71.4 to 193.1 ${\mathrm{mm}}^{2}$) median increase in neo-LVOT surface area. Five (16.7%) patients required permanent pacemaker implantation. Following ASA, TMVR was successfully completed in all cases that were attempted (n = 20). Entire study cohort 30-day mortality was 10% [27]. A retrospective single-center analysis compared procedural characteristics and outcomes in patients who underwent ASA for TMVR (n = 22) *vs*. HCM (n = 80). Pre-emptive ASA prior TMVR was associated with a good safety profile, with no 30-day mortality. However, permanent pacemaker implantation rate tended to be higher in TMVR group, compared to patients with HCM (35% *vs*. 21%; *p* = 0.195) [28]. Candidates for this therapy appears to be patients who have favorable septal perforator anatomy for ASA and do not require immediate TMVR intervention, given the obligatory wait for LV remodeling, which seems to remain the major limitation of alcohol ablation.

Septal radiofrequency ablation is a novel procedure designed to reduce septal thickness in preparation for TMVR. Recently reported in cases where ASA was ineffective or not feasible [15], preemptive septal radiofrequency ablation was conducted under general anesthesia via femoral access, utilizing a 3-dimensional electroanatomical mapping system and intracardiac echocardiography. Both transseptal and retroaortic approaches are possible for left-sided septal ablation. After guiding an externally irrigated ablation catheter to the septal thickening area, ablation is performed for 60 to 90 seconds at each site using saline solutions, until a diminutive electrogram is observed. The initial case series showed promising results in patients with severe MAC, enabling TMVR in all cases [15].

The innovative Septal Scoring Along the Midline Endocardium (SESAME) technique has been developed for “scoring” the septum using a retroaortic approach. In this method, the basal septum is engaged employing a 6-F hockey stick guiding catheter and a guidewire with its tip amputated, along with a microcatheter. Drawing from surgical practices and aiming to reduce potential harm to cardiac conduction tissue, an anterior trajectory, originating below the commissure of the left-right coronary cusps and extended towards the ventricular apex, was opted for. Subsequently, a guidewire designed for chronic total occlusion is swapped and guided along the planned pathway of the basal septum. This guidewire is then captured in the left ventricle using a secondary retroaortic guide. By creating a flying V shape on the guidewire and applying electrocautery while exerting traction on both guiding catheters, the basal septum is effectively lacerated or scored [29]. From this point of view, transcatheter myotomy may offer a septal debulking solution for patients not suitable for ASA. In the first-in-human report, in a patient with concomitant symptomatic obstructive HCM and mitral annular calcification–related mitral stenosis, SESAME procedure expanded the LVOT, alleviated the gradient within the LVOT resulting from the hypertrophied septum, and established sufficient space for MV bioprosthesis implantation [30].

Finally, a kissing balloon inflation approach had been described to sustain LVOT patency, guide positioning and assist in orienting the transcatheter MV bioprosthesis. In this scenario, an aortic valvuloplasty balloon is introduced retrogradely into the LVOT and then inflated under rapid pacing, preceding the inflation of a transcatheter valve in the mitral position. Additionally, the coordinated inflation with the mitral valve prosthesis helps prevent over-flaring of the mitral prosthesis, which could otherwise contribute to mechanical LVOT obstruction [31]. Similarly, Herrmann *et al*. [32] have reported a case where a perfusion balloon was utilized. This method ensures that the LVOT remains open, allowing for blood flow during the process of positioning and deploying the mitral prosthesis. In both cases, no outflow tract obstruction occurred after valve deployment. However, the long-term implications of adopting such a technique remain uncertain and necessitate further experience for proper evaluation.

## 5. Pathophysiology of SAM-Associated MR

SAM of the MV was initially observed as a quite specific finding in HCM, leading to LVOT obstruction. However, among HCM patients with SAM, approximately 25–50% exhibit resting LVOT obstruction. In addition, it is becoming increasingly apparent that SAM is not exclusive to HCM, since it had been described in patients with hypertensive heart disease, perhaps more notable in those with severe untreated hypertension, acute myocardial infarction, especially in the setting of Takotsubo syndrome, and after surgical MV repair, particularly if the distance between MV coaptation and the septum had been surgically diminished through the placement of over-downsized annuloplasty ring.

The exact mechanism behind SAM development remains uncertain. Some investigators proposed that increased flow velocities at the level of an outflow tract distorted by septal hypertrophy might create a Venturi effect, pulling the leaflets towards the septum and obstructing outflow. However, papillary muscle displacement, elongation of the valve leaflet and papillary muscle dyssynchronous contraction may also affect SAM [33]. All these mechanisms can result in disruption of MV leaflet coaptation, giving rise to the development of a posteriorly-directed jet of MR. Interestingly, as demonstrated by Schwammenthal *et al*. [34] in a series of 23 HCM patients, the degree regurgitation did not correlate with the entity obstruction, but increased with increasing mismatch of anterior to posterior leaflet length and decreasing posterior leaflet ability to move anteriorly. Significant SAM-related MR is not uncommon in HCM patients. In a single-center analysis of patients undergoing septal myectomy, at least moderate MR was present in ~40% of the entire study cohort.

## 6. Transcatheter Treatment of SAM-Associated MR

**Fig. 3.**
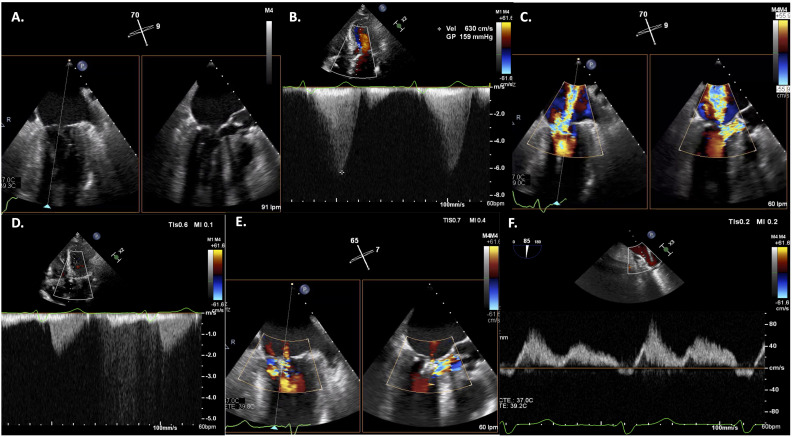
**Mitral transcatheter edge-to-edge repair (M-TEER) in a patient with systolic anterior motion (SAM)-associated mitral regurgitation (MR)**. Transoesophageal echocardiogram showing baseline SAM of the anterior mitral leaflet (AML) (A), leading to severe left ventricular outflow tract (LVOT) obstruction (B) and severe MR (C). After M-TEER with 2 Mitraclip™ implantation, resolution of LVOT obstruction (D), with residual mild MR (E,F).

In patients with SAM-associated MR not deemed for surgical treatment, M-TEER emerged as a possible therapeutic strategy. Targeting a redundant anterior leaflet edge away from the LVOT by merging it with the posterior leaflet aimed to simultaneously (a) improve the degree of regurgitation and (b) decrease outflow tract obstruction gradient in the context of a single procedure, killing two birds with one stone (Fig. 3). In the first-published case series, 3 subjects with obstructive HCM and significant MR in the presence of SAM were referred for MitraClip therapy. After successful and uncomplicated device implantation, significant reduction in basal and provoked LVOT pressure gradients (65 ± 25.5 to 7.7 ± 5 mmHg and 145.3 ± 8.1 to 23.2 ± 7.6 mmHg, respectively) were observed, as well as abolishment of MR with subsequent clinical state improvement [35]. In a single-center experience, 5 obstructive HCM patients with SAM-related MR and drug-refractory heart failure symptoms, underwent M-TEER with single clip positioning at the level of $A_{2}$-$P_{2}$. Procedure resulted in SAM elimination, relief on LVOT obstruction (91 ± 44 mmHg to 12 ± 6 mmHg), associated with hemodynamic improvements in left atrial pressure and cardiac output. One-year follow-up durable absence of SAM and significant MR degree decrease occurred, although high systolic LVOT velocities were observed in 3 of the 5 treated patients, potentially Doppler artifact deriving from clip placement within a narrow LVOT, with downstream pressure recovery [36].

The results proved the concept that SAM is clearly involved in gradient formation, rather than an epiphenomenon in HCM [37].

Advantages of M-TEER rely on (a) minimally invasiveness, with no need creating a septal infarction or altering ventricular structure, thereby reducing the risk of arrhythmias requiring a pacemaker, (b) independence on coronary anatomy and (c) possibility of evaluate hemodynamic effectiveness before permanently securing the clip. However, it is advisable to limit the application of M-TEER to patients who exhibit significant SAM, with a high likelihood of experiencing SAM-related LVOT obstruction. Thus, individuals with extensive septal hypertrophy may not represent the ideal candidates for this treatment strategy, unless it is considered in patients not eligible for ASA or as a bail-out option. Additionally, when considering M-TEER in patients with SAM-related MR after failed surgical annuloplasty, significant increase of transvalvular gradient may represent an issue. Finally, it worth mentioning the current experience is limited to small sample size case series of HCM patients [38]. Further research will clarify the role of M-TEER in treatment of SAM-related MR.

## 7. Conclusions

Two different clinical scenarios may represent the complex interplay between MR and LVOT obstruction: in the first one MR treatment it is responsible for outflow tract gradient increase, meanwhile in the other it represents the solution to LVOT obstruction.

In patients undergoing TMVR, it is necessary to underscore the importance of recognizing LVOT obstruction as a rare yet potentially life-threatening complication, especially since there are limited treatment alternatives following the completion of device deployment. For individuals displaying SAM-related MR, M-TEER might serve as a potentially viable choice, if highly specific anatomical criteria are met.
